# Transcriptome mining of hormonal and floral integrators in the leafless flowers of three cymbidium orchids

**DOI:** 10.3389/fpls.2022.1043099

**Published:** 2022-10-13

**Authors:** Sagheer Ahmad, Kang Yang, Guizhen Chen, Jie Huang, Yang Hao, Song Tu, Yuzhen Zhou, Kai Zhao, Jinliao Chen, Xiaoling Shi, Siren Lan, Zhongjian Liu, Donghui Peng

**Affiliations:** ^1^ Key Laboratory of National Forestry and Grassland Administration for Orchid Conservation and Utilization at College of Landscape Architecture and Art, Fujian Agriculture and Forestry University, Fuzhou, China; ^2^ College of Life Sciences, Fujian Normal University, Fuzhou, China

**Keywords:** leafless orchids, phytohormones, strange phenotypes, transcriptomics, leafless flowering

## Abstract

Flowering is the most studied ornamental trait in orchids where long vegetative phase may span up to three years. Cymbidium orchids produce beautiful flowers with astonishing shapes and pleasant scent. However, an unusually long vegetative phase is a major drawback to their ornamental value. We observed that under certain culture conditions, three cymbidium species (*Cymbidium ensifolium*, *C. goeringii* and *C. sinense*) skipped vegetative growth phase and directly flowered within six months, that could be a breakthrough for future orchids with limited vegetative growth. Hormonal and floral regulators could be the key factors arresting vegetative phase. Therefore, transcriptomic analyses were performed for leafless flowers and normal vegetative leaves to ascertain differentially expressed genes (DEGs) related to hormones (auxin, cytokinin, gibberellin, abscisic acid and ethylene), floral integrators and MADS-box genes. A significant difference of cytokinin and floral regulators was observed among three species as compared to other hormones. The MADS-box genes were significantly expressed in the leafless flowers of *C. sinense* as compared to other species. Among the key floral regulators, *CONSTANS* and *AGAMOUS*-like genes showed the most differential expression in the leafless flowers as compared to leaves where the expression was negligible. However, *CONSTANS* also showed downregulation. Auxin efflux carriers were mainly downregulated in the leafless flowers of *C. ensifolium* and *C. sinense*, while they were upregulated in *C. goeringii*. Moreover, gibberellin and cytokinin genes were also downregulated in *C. ensifolium* and *C. sinense* flowers, while they were upregulated in *C. goeringii*, suggesting that species may vary in their responses. The data mining thus, outsources the valuable information to direct future research on orchids at industrial levels.

## Introduction

Orchids provide the best aesthetic nutrition to mankind. *Cymbidium* is an important genus within the tribe Cymbidieae and contains about 80 species. The species are perennial herbs possessing diverse traits, such as thick and short stem with 4−6 leaves in two whorls and pseudobulbs. The raceme, emerging from the leaf axil of pseudobulblet, is bilaterally symmetrical and bears fragrant flowers (Yang et al., 2021a). Since antiquity, *Cymbidiums* have been grown in ancient China, later spreading to Europe during the Victorian era (Hew, 2001). Continuous diversification of perianth color and floral pattern, and a unique fragrance make them the most popular orchids (Kim et al., 2016; Ramya et al., 2019). So far, the Royal Horticultural Society has registered more than 150,000 commercial *Cymbidium* hybrids (Yang et al., 2021a). Therefore, *Cymbidium* becomes the ideal taxon to study the flower development and the morphological evolution of orchids (Motomura et al., 2010).

However, the long waited flowering after a vegetative growth of more than two years makes their value less economic (Ahmad et al., 2021a; Ahmad et al., 2022a). Thus, reducing vegetative growth is central to studies involving orchids. After seed germination, protocorm is established, the first stage of embryo development, to obtain nutrition for the developing plantlet through symbiotic relationships with fungus. The plant embryo develops into a miniature sporophyte after fertilization (Fang et al., 2016). The embryogenesis proceeds through two phases: morphogenesis and maturation (Bentsink and Koornneef, 2008; Braybrook and Harada, 2008). During morphogenesis, organization occurs for different body components, such as apical-basal polarity, functionally organized domains, cell differentiation and tissue specification (Steeves and Sussex, 1989). Genetic regulators play significant roles in the regulation of axis polarity and division plane (Jeong et al., 2012; Ueda and Laux, 2012).

Phytohormones has been considered as important regulators of orchid flowering (Goh and Yang, 1978). Auxin plays significant roles as morphogen (Bhalerao and Bennett, 2003; Benková et al., 2009; Möller and Weijers, 2009; Lau et al., 2011; Finet and Jaillais, 2012). Its concentration gradient across plant body provides cues for tissue specification (Zoulias et al., 2019). Cytokinin is plant growth activator. Synthetic cytokinin application promotes flowering in *Dendrobium* and *Phalaenopsis* orchids. Moreover, cytokinin applied in combination with gibberellin (GA) enhances flowering (Hew and Clifford, 1993). GAs are well-known regulators of important developmental processes, such as flowering time (Ding et al., 2013; Hyun et al., 2016). Abscisic acid (ABA) coordinates flowering time and bud break (Wang et al., 2013b; Shu et al., 2016). Fluctuations in ethylene levels can either promote or delay flowering in many species, such as pineapple (Trusov and Botella, 2006), roses (Meng et al., 2014), rice (Wang et al., 2013a) and Arabidopsis (Achard et al., 2007). For example, accumulation of ethylene triggers *TEOSINTE BRANCHED 1/CYCLOIDEA/PCF* (*TCP*) genes, which ultimately inhibits the progression of cell cycle (Dubois et al., 2018). Moreover, ethylene induces the expression of *ERF5* and *ERF6* and *EFR6* induces gibberellic acid interacting enzyme *GA2-OX6*, leading to reduced bioactive GA levels and accumulation of DELLA proteins (Skirycz et al., 2011; Dubois et al., 2013; Meng et al., 2013). Thus, hormones could be the key to stimulate altered growth cycles leading to leafless flowering in orchids.

A number of MADS-box genes have been identified in orchids with their significant roles in flowering, and advanced models have been suggested for orchid flower development (Aceto and Gaudio, 2011; Yang et al., 2017). Studies have documented the functional characterization of a number of genes related to flowering in orchids, such as *FT* homologs and *FD*, the gene encoding *FT* interacting protein, in *Phalaenopsis aphrodite, Oncidium* Gower Ramsey, and *Dendrobium nobile*. Moreover, the *LEAFY* and *CONSTANS-like genes* have been identified in *Phalaenopsis aphrodite*; while the genes for co-regulated transcription factors such as *SQUAMOSA promoter binding-like genes* (*SPL-like*) and *CINCINNATA-like* (*TCP-like*) have also been found in orchids (Hou and Yang, 2009; Chou et al., 2013; Lin et al., 2013; Jang, 2015; Lin et al., 2016; Liu et al., 2016). The MADS-box gene *AP1*/*AGL9* is involved in the regulation of floral transition and flower organ development (Theißen, 2001). The *AP1*/*SQUA*-like genes play key roles in meristem identity determination (Chen et al., 2007). A number of *SEP* (*SEPALLATA*)-like genes play roles in orchid floral structure formation (Salemme et al., 2013). *AP1* acts as a hub between *SOC1* and *SVP*, both determining the floral organ identity (Honma and Goto, 2001).

Surprisingly, we observed leafless flowering in three orchid species in closed environment chambers. The vegetative growth was absolutely bypassed by the developing protocorms, directly flowering without leaves. It offsets a new direction on research for rapid orchid flowering with limited vegetative growth. Transcriptome analysis was performed to compare leafless flowers and healthy leaves for *C. ensifolium*, *C. goeringii* and *C. sinense*. Important hormonal and flowering regulators were mined, which may serve as building blocks to plan functional studies for rapid orchids.

## Materials and methods

### Plant materials and growth conditions

The *Cymbidium* species (*C. ensifolium*, *C. goeringii* and *C. sinense*) were grown in the tissue culture facility of Fujian Agriculture and Forestry University. The media contained NAA (0.5 mg L^-1^), 6-BA (8.0 mg L^-1^), activated carbon (1.5 g L^-1^), sugar (35 g L^-1^) and agar (7.0 g L^-1^). The growth temperature was set to 26 ± 2 °C at a light intensity of 2,500-3,000 Lx and the photoperiod was 12 h/day. After about six months, the leafless flowers were produced, and the fully opened flowers were collected for RNA Sequencing. The leaf samples were obtained from normally growing species as a reference.

### RNA-seq library preparation and sequencing

A total of six tissues in replicates (18 samples) were used for RNA extraction using TaKaRa RNA extraction kit. The cDNA libraries were produced using the total RNA. The mRNA was obtained using the Oligotex Midi Kit (Qiagen, Germany) and the quality and quantity of mRNA was checked on Nano-Drop spectrophotometer (Thermo Fisher Scientific, USA). Then, the cDNA libraries were prepared following the Illumina protocol and the library products were evaluated through Qubit^®^2.0 and Agilent 2200 TapeStation (Life Technologies, USA). The purified products were diluted to 10 pM for the generation of *in situ* clusters through HiSeq2500 pair-end flow cells and pair-end sequencing (2 × 100). In the end, reference-based sequencing was carried out by using the reference genome of each species. The gene expression was calculated based on FPKM (fragments per kilobase per transcript per million mapped reads).

### Functional annotation

Publically available datasets were used to map the assembled genes. The mapping was done the BLASTX program (threshold E-value ≤ 10^-5^) for KEGG (Kyoto Encyclopedia of Genes and Genomes), GO (Gene Ontology), KO (KEGG ortholog) and NR (non-redundant) annotations. The KEGG and GO annotations results were classified into pathways and functional categories the R software for phyper function (https://en.wikipedia.org/wiki/Hypergeometric_distribution). The false discovery rate (FDR) was used to calculate corrected *p* values and the terms with *q* value ≤ 0.05 were recognized a significantly enriched.

### Differentially expressed genes

The clean reads were aligned using the Bowtie2 software and their expression levels were ascertained using RSEM (v1.2.8) with default parameters. Then, the DEGs were obtained using R software for DEGseq package (v1.10.1). The significantly differential genes were filtered at a threshold *p*-value < 0.001 and the log2FC > 1.

### Identification of hormone and flowering related DEGs

The DEGs were filtered using keywords, such as flowering, MADS, auxin, cytokinin, gibberellin, abscisic acid, and ethylene to identify genes related to flowering and hormonal regulation. Those with significant difference were selected for drawing heatmaps using TBtools.

Similarly, the DEGs were filtered with biological processes annotations related to the regulation of hormones, flower development, flowering time, biological clocks and other related pathways.

### Statistical analysis

The transcriptomic data was analyzed using the Pearson correlation coefficient, log2 fold change and threshold *p* and *q* values for DEGs.

## Results

### Transcriptome data

For *C. ensifolium*, each sample produced an average of 6.37 Gb of data. The average alignment rate of the sample compared to the genome was 86.60%, and the average alignment rate of the compared gene set was 66.55%. The predicted new genes were 5,139 and the total number of expressed genes was 26,227, of which the known genes were 21,642 and 4,585 were predicted new genes (**Supplementary Tables 1**–**3**).

For *C. goeringii*, each sample produced an average of 6.59 Gb of data. The average alignment rate of the sample compared to the genome was 78.44%, and the average alignment rate of the compared gene set was 60.53%. The predicted new genes were 4,400 and the total number of expressed genes detected was 30,992, of which the known genes were 26,763 and 4,229 were predicted novel genes (**Supplementary Tables 4**–**6**).

In the case of *C. sinense*, each sample produced an average of 6.62 Gb of data. The average alignment rate of the sample compared to the genome was 82.54%, and the average alignment rate of the compared gene set was 61.90%. The predicted new genes were 3,909 and the total number of expressed genes detected was 27,282, of which the known genes were 23,457 and 3,825 were predicted new genes (**Supplementary Tables 7**–**9**).

### Expression analysis and comparison among three species

The expressions of leafless flowers were compared with the leaves for three orchid species. The empirical cutoff gene values were sued with positive expressions. The distribution of FPKM values is presented as boxplots, showing the uniform median and quartile distribution of DEG expression between samples of each species (**Supplementary Figure 1**).

*C. goeringii* showed the highest number of upregulated and downregulate genes (4089) (**Supplementary Figure 2**), as compared to *C. ensifolium* (3414) and *C. sinense* and *C. sinense* (2807) (**Supplementary Figure 2**). *C. ensifolium* showed the highest number of flower specific DEGs than other two species.

### Gene annotation analyses

The GO and KEGG annotations were obtained for each species. The GO biological process annotation of *C. ensifolium* shows that the highest number of genes were enriched in metabolic and cellular processes (**Supplementary Figure 3**). In cellular components, the maximum number of genes were obtained in cellular anatomical entities and intracellular components. The most enriched molecular functions were shown in catalytic activity and binding. The GO annotations of *C. goeringii* were similar to *C. ensifolium* and *C. sinense*. However, the number of genes were less in *C. sinense* as compared to other two species (**Supplementary Figure 3**).

For the KEGG pathway enrichment, plant hormone signal transduction pathway was the highly enriched pathway among other pathways, including phenylpropanoid biosynthesis and plant pathogen interaction pathway (**Supplementary Figure 4**).

### Flowering and hormone related GO biological processes

**Figure 1:**
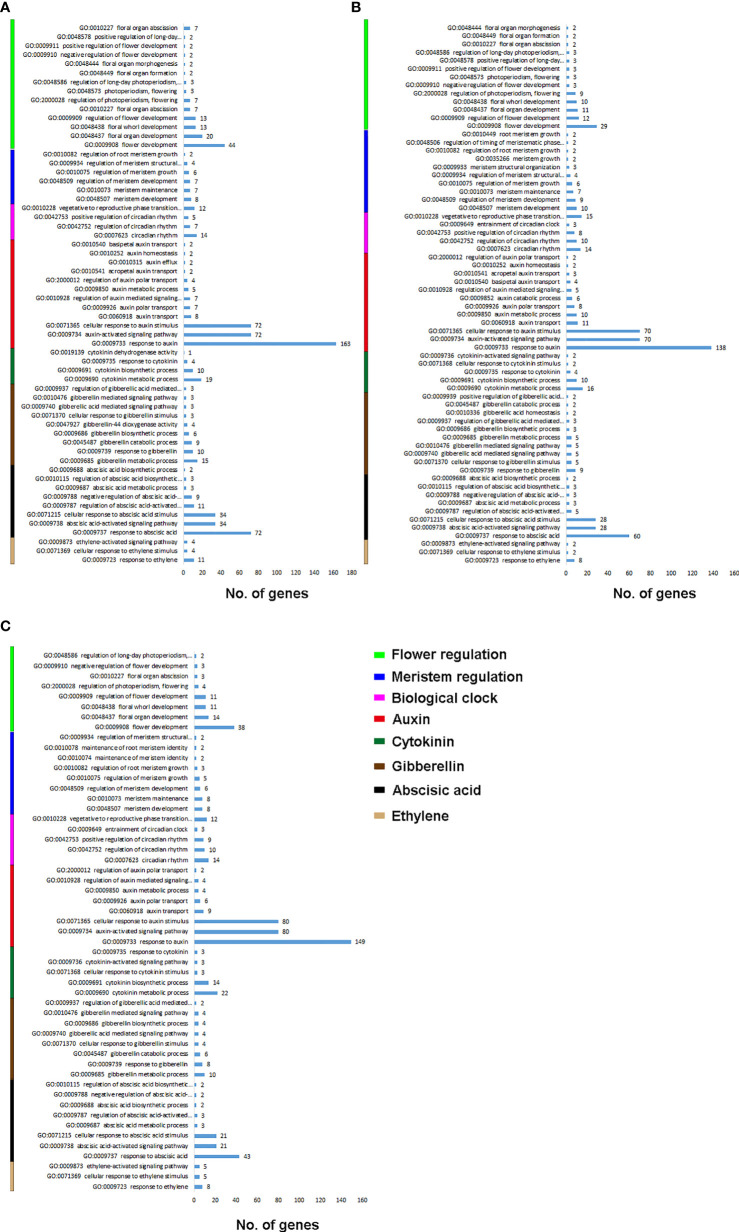
Flowering and hormone related biological processes for C. ensifolium **(A)**, C. goeringii **(B)** and C. sinense **(C)**.

We filtered the GO biological processes for flowering and hormonal regulation (**Figure 1**). **Figure 1A** shows the biological process enrichment for *C. ensifolium*. Flower development (GO:0009908) was enriched by the highest number of genes (44), followed by floral organ development (GO:0048437) and flora whorl development (GO:0048438). The key biological processes for meristem activity included meristem development (GO:0048507) and meristem maintenance (GO:0010073). For biological clock regulation, the highest number of genes were observed for vegetative to reproductive phase change (GO: 0010228) and circadian rhythm (GO:0007623). The highest number of genes were enriched in response to auxin (GO:0009733), auxin-activated signaling pathway (GO:0009734) and cellular response to auxin (GO:0071365). The other key hormone-related biological processes included cytokinin metabolic process (GO:0009690), gibberellin metabolic process (GO:0009685), response to ABA (GO:0009737), ABA-activated signaling pathway (GO:0009738), cellular response to ABA stimulus (GO:0071215), and response to ethylene (GO:0009723).

For *C. goeringii*, more number of genes were enriched for flowering and auxin regulation as compared to rest of the integrators (**Figure 1B**). The highly enriched biological processes for flowering included flower development (GO:0009908), regulation of flower development (GO:0009909), floral organ development (GO:004837) and floral whorl development (GO:0048438). Meristem development (GO:0048507) and meristem maintenance (GO:0010073) were the main biological process for meristem activity. Vegetative to reproductive phase transition (GO: 0010228) and circadian rhythm (GO:0007623) were mainly involved in biological clock regulation. Response to auxin (GO:0009733), auxin-activated signaling pathway (GO:0009734) and cellular response to auxin (GO:0071365) were the highly enriched auxin-related pathways. Among the other highly enriched biological process for hormones included response to ABA (GO:0009737) enriched by 60 genes, ABA-activated signaling pathway (GO:0009738) and cellular response to ABA stimulus (GO:0071215)

For *C. sinense*, the flowering related biological process enrichment was less as compared to other two species (**Figure 1C**). The highly enriched biological processes for flowering included flower development (GO:0009908), and floral organ development (GO:004837) and floral whorl development (GO:0048438). Meristem enrichment was also less than other two species, including mainly meristem development (GO:0048507) and meristem maintenance (GO:0010073). Vegetative to reproductive phase transition (GO: 0010228) was the key biological clock regulation. Interestingly, the auxin regulatory processes were highly enriched in *C. sinense* as compared to *C. ensifolium* and *C. goeringii*. Response to auxin (GO:0009733) was enriched in 149 genes, auxin-activated signaling pathway (GO:0009734) in 80 genes and cellular response to auxin (GO:0071365) was enriched in 80 genes. The other key hormone-related biological processes included cytokinin metabolic process (GO:0009690), gibberellin metabolic process (GO:0009685), response to ABA (GO:0009737) enriched by 43 genes, ABA-activated signaling pathway (GO:0009738), cellular response to ABA stimulus (GO:0071215), and response to ethylene (GO:0009723).

### Auxin regulators

**Figure 2:**
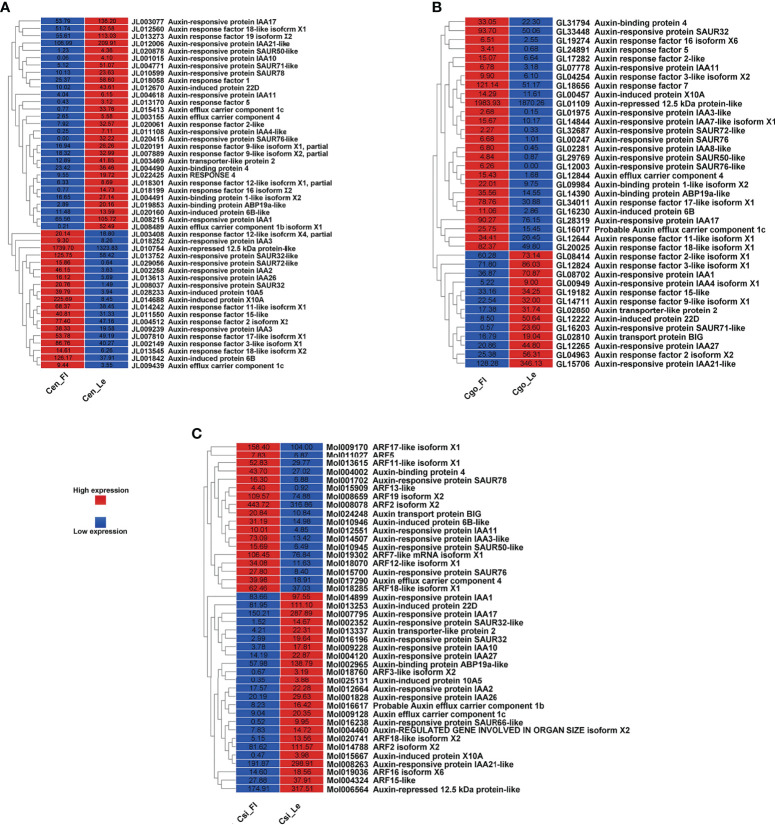
Auxin pathway genes for C ensifolium **(A)**, C goeringii **(B)** and C sinense **(C)**.

The auxin regulation was mainly manifested as auxin responsive proteins, auxin response factors (ARFs) and auxin transport proteins (**Figure 2**). More number of downregulated auxin-related genes can be seen in *C. ensifolium* as compare to other species (**Figure 2A**). Moreover, the number of auxin-related genes was high in *C. ensifolium* as compared to other species. The downregulated genes were mainly related to auxin responsive proteins and auxin binding proteins, while the upregulated genes were mainly related to ARFs. Four auxin efflux carrier components were found and only one of them was upregulated while other three were downregulated. The auxin response protein SAUR71-like and SAUR76-like, auxin efflux carrier component 1c and 1b, auxin response factor 16, and auxin binding protein ABP19a-like were the most downregulated genes in the leafless flowers. The most upregulated included auxin responsive protein IAA2, auxin responsive proteins SAUR32 and SARU72-like, and auxin induced protein 10A5.

For *C. goeringii*, the auxin regulatory genes were more upregulated than downregulated genes (**Figure 2B**). The upregulated included a large proportion of auxin responsive proteins, while the downregulated contained mostly the ARFs. Two auxin efflux carrier components were upregulated in the leafless flowers of C. goeringii (**Figure 2B**). SAUR71-like was also among the most downregulated gene flowers, while the upregulated included IAA8, and auxin response factor ARF5.

More number of downregulated auxin genes were observed in *C. sinense*, including mainly the auxin responsive proteins and ARFs (**Figure 2C**). Two components of auxin efflux carriers were observed: one was upregulated and the other was downregulated in the leafless flowers. AFR13-like was the most upregulated gene, while SARU32-like, SAUR66-like and IAA10 were the most downregulated genes (**Figure 2C**).

**Figure 3:**
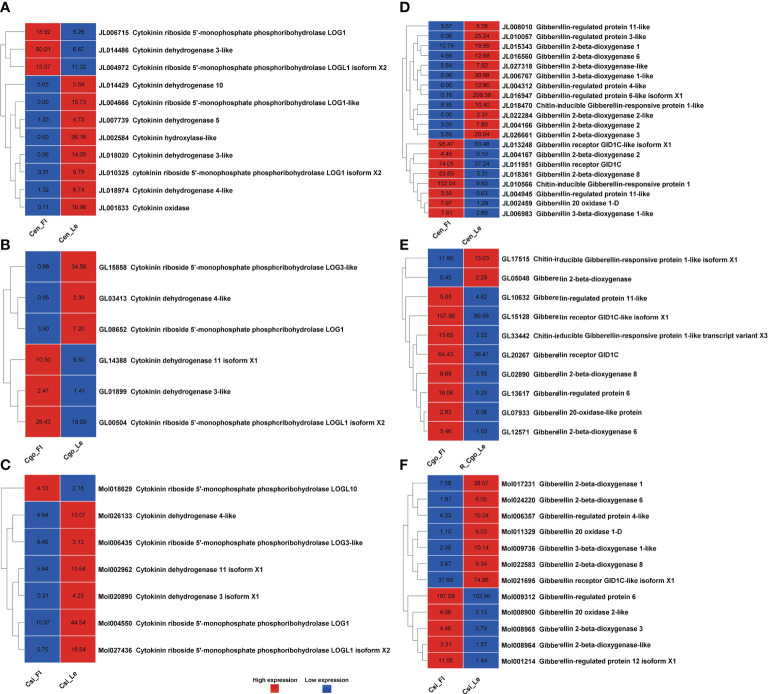
Cytokinin pathway genes for C. ensifolium **(A)**, C. goeringii **(B)** and C. sinense **(C)**; and gibberellin pathway genes for C. ensifolium **(D)**, C. goeringii **(E)** and C. sinense **(F)**.

### Floral accelerators: cytokinins and gibberellins

Cytokinin regulatory genes were much less than other hormones (**Figures 3A–C**). In *C. ensifolium* (**Figure 3A**) and *C. sinense* (**Figure 3C**), the cytokinin-related genes were downregulated in the leafless flowers. Only 6 cytokinin genes were observed in *C. goeringii* (**Figure 3B**), including 3 upregulated and 3 downregulated genes. Four *LOG1* genes were found in *C. ensifolium*; two were upregulated and two were downregulated. Three *LOG* genes were found in *C. goeringii*; two were downregulated and one was upregulated. Only one *LOG* gene was upregulated in *C. sinense* and the remaining three were downregulated.

A total of 20 gibberellin related DEGs were found in *C. ensifolium* (**Figure 3D**), including 12 upregulated and 8 downregulated. Most of these genes regulate various steps of gibberellin biosynthesis. Gibberellin regulated protein 3-like, Gibberellin regulated protein 4-like and gibberellin 3-beta-dioxygense 6 were the highly downregulated genes in the leafless flowers, while gibberellin 20 oxidase 1-4 and gibberellin 2-beta dioxygenase 8 were the highly upregulated gene. Out of 10 highly expressed gibberellin genes, 8 were upregulated and 2 were downregulated in *C. goeringii* (**Figure 3E**). Among the upregulated proteins, gibberellin regulated protein showed the highest difference in the leafless flowers as compared to leaves. In *C. sinense*, 12 DEGs were found related to gibberellin, including 7 downregulated and 5 upregulated DEGs (**Figure 3F**). Here, gibberellin 20 oxidase 1-D was the most significantly downregulated and the gibberellin 2-beta dioxygenase 3 was the most upregulated gene. Gibberellin regulation was significantly different in *C. goeringii* as compared to *C. ensifolium* and *C. sinense*.

### Floral inhibitors: ABA and ethylene

**Figure 4:**
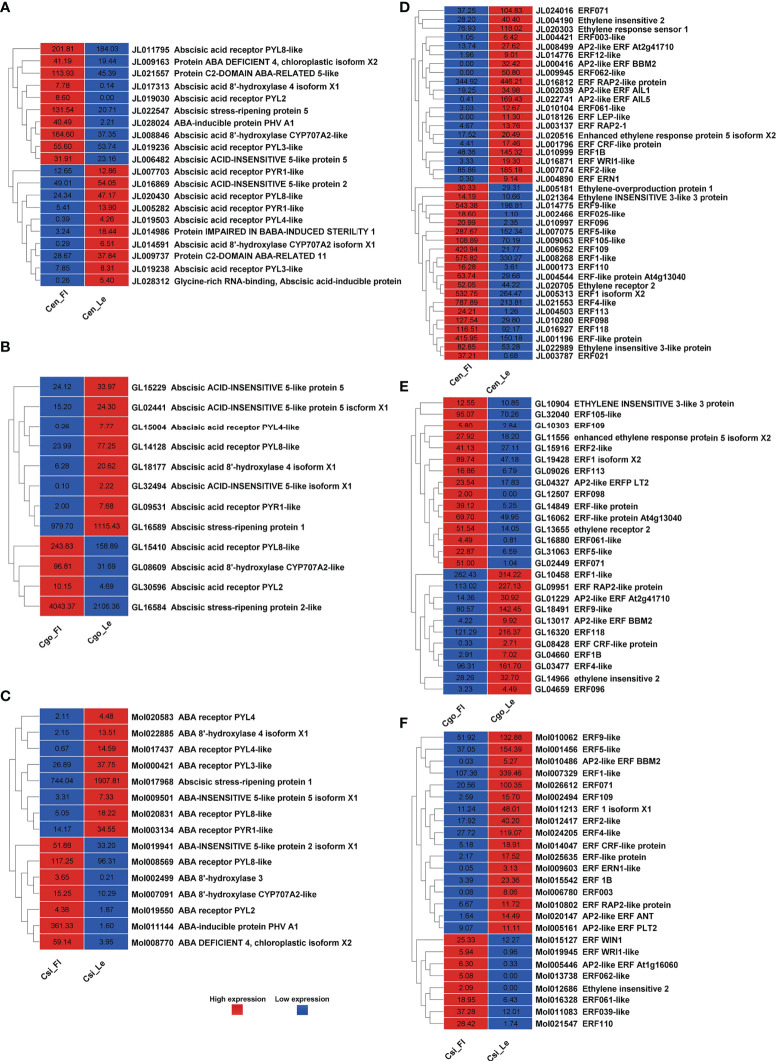
ABA pathway genes for C. ensifolium **(A)**, C. goeringii **(B)** and C. sinense **(C)**; and ethylene pathway genes for C. ensifolium **(D)**, C. goeringii **(E)** and C. sinense **(F)**.

An equal number of upregulated and downregulated ABA regulators were found in *C. ensifolium* (**Figure 4A**), with a total of 20 DEGs, higher than other two species. *PYL2* was only expressed in leafless flowers, while *PYL4* and *CYP707A2* showed significant downregulation. Some genes appeared in multiple isoforms. *C. goeringii* showed 8 downregulated and 4 upregulated DEGs, which also appeared in isoforms with both upregulated and downregulated forms (**Figure 4B**). Here *PYL4* was mainly downregulated. Out of 15 highly differential ABA genes in *C. sinense*, 8 were downregulated and 7 were upregulated in the leafless flowers (**Figure 4C**). *PYL4* showed the most differential expression in healthy leaf as compared to leafless flower, while ABA-inducible protein PHV expressed at extremely high level in flowers as compared to leaf.

After auxin, the ethylene related DEGs were the most abundant among hormone regulators in three *Cymbidium* species (**Figures 4D–F**). In *C. ensifolium*, 40 DEGs showed differential expression between leafless flowers and leaves, with equally up- and down-regulated genes (**Figure 4D**). In *C. goeringii*, 15 DEGs were upregulated and 11 were downregulated in the ethylene pathway (**Figure 4E**). Most of the genes were ethylene response factors (ERFs). However, ethylene regulation in the leafless flowers of *C. sinense* was much different than other two species (**Figure 4F**). Here, 17 ethylene-related DEGs were downregulated and 8 were upregulated in the leafless flowers as compared to healthy leaves.

### Flowering pathways and MADS-box genes

**Figure 5:**
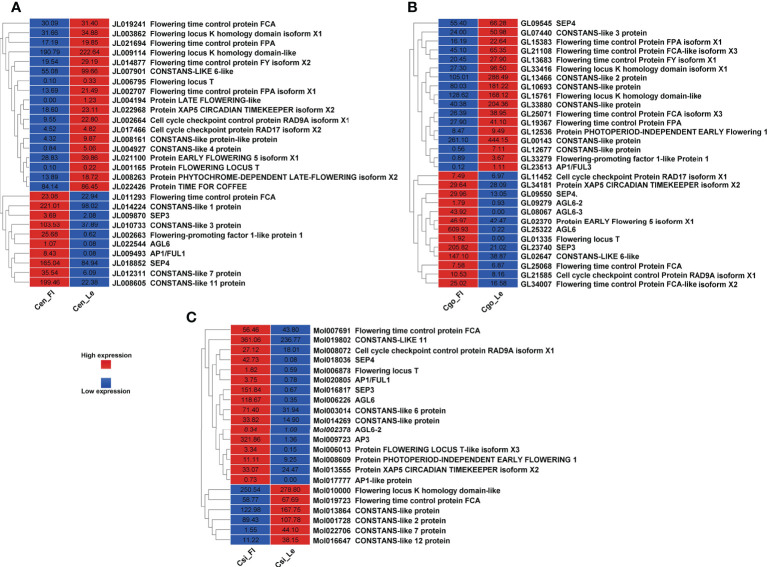
Flowering related genes for C. ensifolium **(A)**, C. goeringii **(B)** and C. sinense **(C)**.

A significant differential expression of floral integrators was found for *C. ensifolium* (**Figure 5A**) and *C. sinense* (**Figure 5C**) as compared to *C. goeringii* (**Figure 5B**) where almost equal number of upregulated and downregulated DEGs were observed. Among the 28 flowering related DEGs in C. ensifolium, 18 showed less expression in leafless flowers and 10 showed high expression (**Figure 5A**). The floral integrators were mainly related to flowering time control and cell cycle activities. The most prominent expression differences were shown by *CONSTANS* and *AGLs* as compared to other floral regulators. Out of 30 DEGs in *C. goeringii*, 13 showed high expression and 17 showed low expression in leafless flowers (**Figure 5B**). 22 DEGs were expressed in *C. sinense*; however, contrary to other two species, here 16 genes were upregulated and only 6 were downregulated (**Figure 5C**).

**Figure 6:**
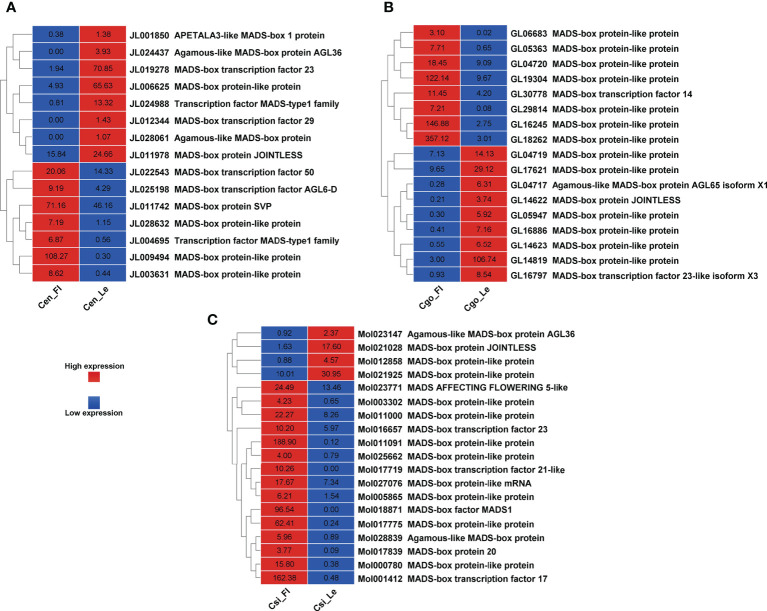
MADS-box genes for C. ensifolium **(A)**, C. goeringii **(B)** and C. sinense **(C)**.

A number of MADS-box genes were also found with contrasting expressions in three species (**Figure 6**). The MADS-box genes were almost equally upregulated and downregulated in *C. ensifolium* (**Figure 6A**) *and C. goeringii* (**Figure 6B**). However, the highest number of MADS-box (19) were observed in *C. sinense* (**Figure 6C**) with 4 upregulated and 15 downregulated DEGs.

## Discussion

*Cymbidium* orchids are thought to be the most precious and beautiful flowers with enormous economic, ornamental and aesthetic values. *Cymbidium sinense*, *C. ensifolium* and *C. goeringii* are representative orchids with versatile and scented flowers. However, an extended vegetative phase of 2-3 years puts a major hurdle in their market success. Recently, studies have been focusing the flowering time manipulation with no practical output. There is an urgent need to devise strategies for the development of new orchid varieties with limited vegetative phase and controlled flowering time. Our study found that the above mentioned three orchids escaped vegetative phase and directly produced leafless flowers through protocorms in the controlled environment. This abnormal flowering pattern lead to transcriptome analysis in order to find differentially expressed gene sets between leafless flowers and healthy leaves. We mainly concentrated on DEGs related to hormone regulation and flower integration.

Floral organ plan foundation needs multiple harmonized spatiotemporal courses, including the perception of positional information that stipulates floral organ founder cells and floral meristem, coordinated organ outgrowth associated with the generation and maintenance of inter-whorl and inter-organ boundaries, and the meristem activity termination. Auxin assimilates the gene regulatory networks to control these processes, and play an instructive part in the tissue-specific biosynthesis and transport to fashion local maxima, perspicacity, and signaling (Cucinotta et al., 2021). ARFs (AUXIN RESPONSE FACTORs) are imperative to auxin transport between bud and stem during bud break (Hayashi, 2012). Floral bud is regulated by endogenous hormones, including promoters, such as IAA, CK, and GA3 and inhibitors, such as ABA, ethylene and JA (Beveridge et al., 2009; Müller and Leyser, 2011; Kebrom et al., 2013; Barbier et al., 2015; Wang and Wang, 2015; Yuan et al., 2015). A continuous flower orchid *Arundina graminifolia* transcriptome contained a large proportion of hormone-related genes, such as auxin, gibberellin, and ABA (Ahmad et al., 2021a). In *Paphiopedilum callosum* orchid, the GA_3_ application upregulates floral homeotic genes, such as *AP3* and *SEP* in the floral buds (Yin et al., 2022), thereby promoting continuous flowering. Our data showed high gene enrichment for phytohormones. GO annotation shows that most of the genes were enriched in the auxin pathway, such as response to auxin (GO:0009733) was shown by 163 genes, and auxin-activated signaling pathway (GO:0009734), cellular response to auxin (GO:0071365) and response to abscisic acid (GO:0009737) by 72 genes, respectively (**Figure 1A**) in *C. ensifolium*. Almost similar enrichments were observed in other two species, suggesting that hormonal pathways may share significant part in abnormal flowering phenotype. Auxin pathway genes were more downregulated in *C. ensifolium* and *C. sinense*; while *C. goeringii* transcriptome showed more upregulated DEGs (**Figure 2**). In C. ensifolium, auxin response protein SAUR71-like and SAUR76-like, auxin efflux carrier component 1c and 1b, auxin response factor 16, and auxin binding protein ABP19a-like were the most downregulated genes in the leafless flowers. The most upregulated included auxin responsive protein IAA2, auxin responsive proteins SAUR32 and SARU72-like, and auxin induced protein 10A5 (**Figure 2A**). SAUR71-like was also among the most downregulated gene in *C. goeringii* flowers, while the upregulated included IAA8, and auxin response factor ARF5 (**Figure 2B**). *C. sinense* showed AFR13-like as the most upregulated gene, while SARU32-like, SAUR66-like and IAA10 were the most downregulated genes (**Figure 2C**). A significant cytokinin downregulation can be seen in *C. ensifolium* and *C. sinense*, suggesting that cell activities may remain limited during abnormal floral bud growth (**Figure 3**), which also correlated with limited gibberellin activity in both the species. ABA was mainly downregulated in all the species (**Figure 4**); while ethylene was more downregulated in C. sinense than other two species. Ethylene regulates DELLA proteins in the gibberellin pathway (Meng et al., 2013). It regulates flower bud development and flower formation in bulbous plants (De Munk and Duineveld, 1986). Ethylene plays a crucial role in the regulation of flower senescence (Dar et al., 2021), suggesting it an inhibitor. However, the exact role of ethylene in flower bud development needs extensive research.

*SVP* (*SHORT VEGETATIVE PHASE*), a flowering time regulator, interacts with *TCP* TFs during bud bread (Singh et al., 2019). The *C. goeringii SVP* gene interacts with *CgAP1* and *CgSOC1* in the regulation of flower development (Yang et al., 2019). *AP1* performs a hub role between *SOC1* and *SVP*, which are famous proteins determining floral organ identity (Honma and Goto, 2001). *SVP* interacts with *FLC* and *FLM*, leading to repression of *FT* during temperature and photoperiod pathways for flowering regulation (Fujiwara et al., 2008; Gregis et al., 2009; Tao et al., 2012). It also regulates hormones, such as GA and ABA during the bud break (Singh et al., 2018). *CONSTANS* (*CO*) are the zinc finger TFs and involve flowering time regulation (Ordoñez-Herrera et al., 2018). We found all these important floral and hormonal integrators with significant expression difference between leafless flowers and healthy leaves (**Figures 5**, **6**). The flowering-related genes were comparatively downregulated in *C. ensifolium* and *C. goeringii* as compared to *C. sinense*, where most of the genes were upregulated (**Figure 5**), suggesting that species may differ in their responses for genetic regulators. *CONSTANS* were the most significantly downregulated flowering genes in three species, although they also showed upregulation, which was less differential than downregulation (**Figure 5**). The other significantly upregulated flowering genes included the hub genes known for multiple regulatory pathways for floral integration, such as *SEP*, *AP1*, *AGL6*, *FT* and *FCA*.

Our recent studies have found a number of TFs related to flowering regulation in orchids, especially the Arundina graminifolia (Ahmad et al., 2020; Ahmad et al., 2021a; Ahmad et al., 2022a). Our study also showed a number of MADS-box and zinc finger TFs with significant difference in the leafless flowers and healthy leaves of three *Cymbidium* species (**Figures 5**, **6**), which are known floral regulators in orchids (Teo et al., 2019; Valoroso et al., 2019; Ahmad et al., 2021b; Chen et al., 2021; Yang et al., 2021b; Ahmad et al., 2022a; Ahmad et al., 2022b). However, more flowering related genes and TFs were upregulated in *C. sinense* as compared to other two species (**Figure 5**), suggesting that *C. sinense* may have different body plans than other *Cymbidium* orchids.

In short, the production of leafless flowers in *Cymbidium* orchids provides a robust source to genetically engineer new orchid varieties with limited vegetative phase. Our transcriptome data is enriched with a number of hormonal and floral regulators. *CONSTANS*, *SPLs*, *AP* and *SEPs* showed distinct expression differences between leafless flowers and healthy leaves. Among the hormones, auxin and ethylene contained the most abundant genes. The data is a raw material to devise future research for transgenic orchids.

## Conclusions

**Figure 7:**
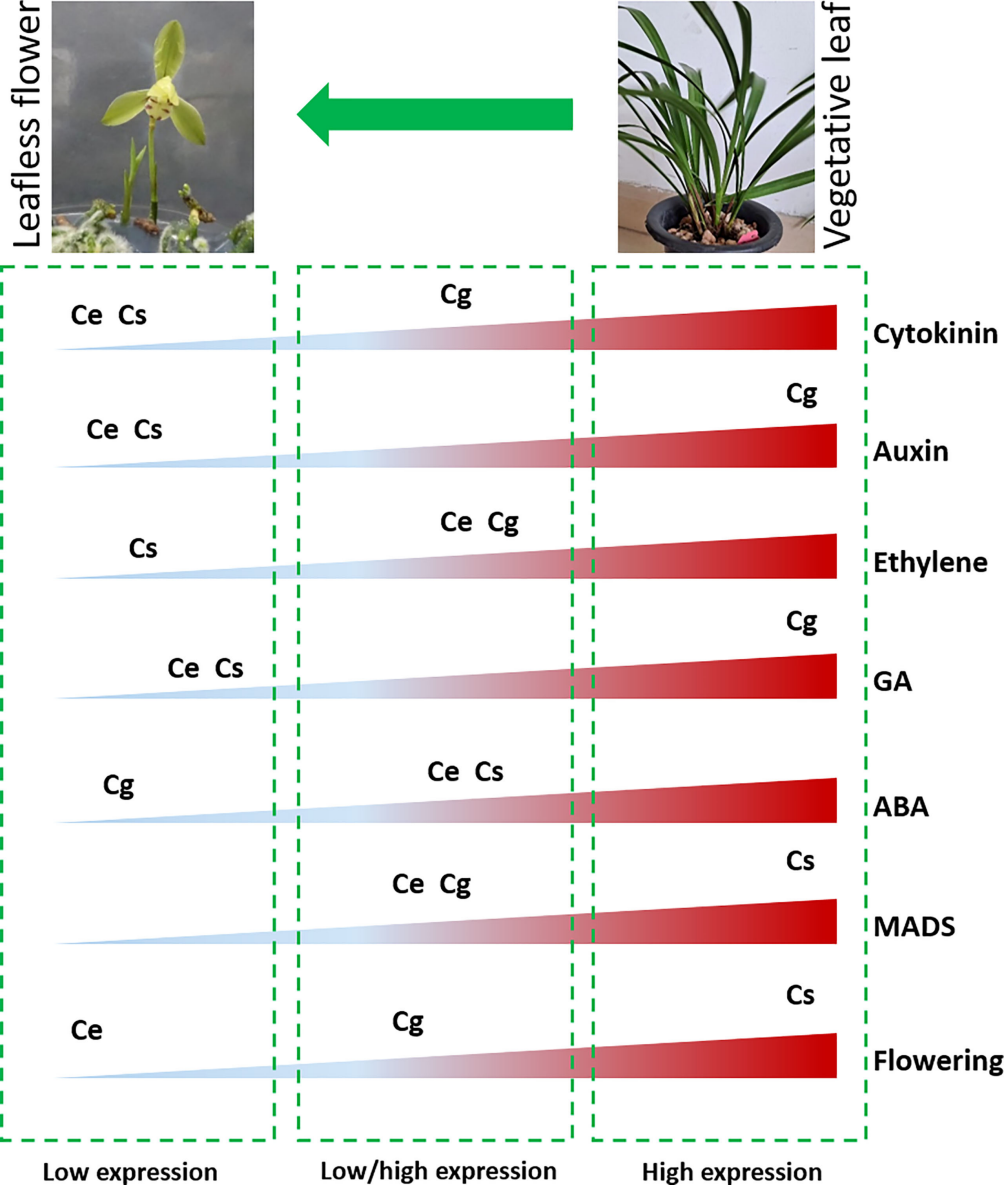
Overview of differential abundance of gene expression for flowering and hormonal pathway genes between leafless flowers and healthy leaves (Ce: C. ensifolium; Cg: C. goeringii; Cs: C. sinense).

The subject matter is that, a strange leafless flowering phenotype was observed in three *Cymbidium* species in controlled environment (**Figure 7B**), wherein no leaf growth was observed. The flowers appeared in six months, which is astonishing when comparing normal growth cycle of more than 2 years for most of the orchids. The transcriptome data mined a number of hormonal and floral regulatory genes, which were differentially expressed in the leafless flowers and healthy leaves of three species (**Figure 7**). Auxin and gibberellin related genes showed high expression in C. goeringii, while the flowering genes were highly expressed in C. sinense (**Figure 7**). Ethylene and ABA related genes were mainly downregulated. *CONSTANS* for flower regulation, auxin efflux carriers, *LOGs* in the cytokinin biosynthesis pathway, gibberellin 20 oxidase in the GA biosynthesis pathway, and *PYLs* in the ABA pathway could be the key outputs of this study. This output provides enough genetic information to build future functional ground for reduced vegetative phase alteration in precious orchid species.

## Data availability statement

The transcriptome data described in this article has been submitted to “The National Genomics Data Center” (NGDC, https://ngdc.cncb.ac.cn) under accession number: PRJCA009885.

## Author contributions

SA: Conceptualization, Writing-original draft; KY: Data curation; GC: Data curation, software; JH: Investigation; YH: Software; ST: Data curation; YZ: Visualization, Investigation, editing; KZ: Data curation, conceptualization; JC: Data curation, software; XS: Investigation; SL: Software, editing; ZL: Supervision, Conceptualization, Funding acquisition; DP: Supervision, Conceptualization, Funding acquisition, Writing-Reviewing and editing. All authors contributed to the article and approved the submitted version.

## Funding

This work was supported by The National Natural Science Foundation of China (32071815); The National Key Research and Development Program of China (2019YFD1001000); The National Key Research and Development Program of China (2018YFD1000401); The Innovation and Application Engineering Technology Research Center of Ornamental Plant Germplasm Resources in Fujian Province (115-PTJH16005) and National Natural Science Foundation of China (32101583).

## Acknowledgments

We are thankful to funding agencies for funding support.

## Conflict of interest

The authors declare that the research was conducted in the absence of any commercial or financial relationships that could be construed as a potential conflict of interest.

## Publisher’s note

All claims expressed in this article are solely those of the authors and do not necessarily represent those of their affiliated organizations, or those of the publisher, the editors and the reviewers. Any product that may be evaluated in this article, or claim that may be made by its manufacturer, is not guaranteed or endorsed by the publisher.
