# Supplementary material for: Transcriptome mining of hormonal and floral integrators in the leafless flowers of three cymbidium orchids
Source: Front Plant Sci. 2022 Oct 13;13:1043099. doi: 10.3389/fpls.2022.1043099 (PMC9608508; doi:10.3389/fpls.2022.1043099)

**Supplementary figure 1** The boxplot distribution of gene expression the two tissues of each species. The X-axis shows the sample name; the Y-axis represents log10(FPKM+1). The boxplot of each area shows five statistics (from top to bottom are the upper limit, upper quartile, median, lower quartile, respectively).


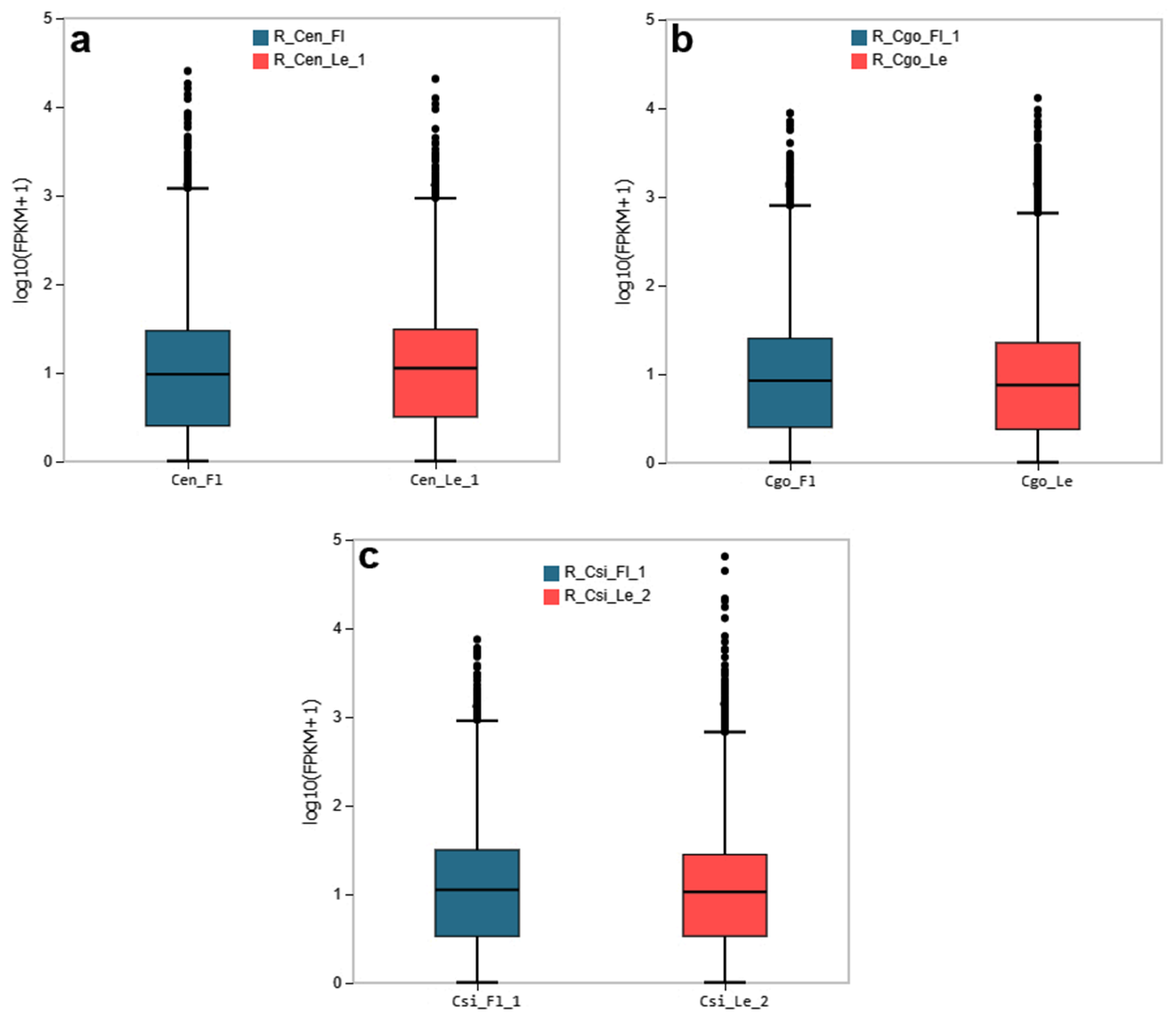


**Supplementary figure 2** Individual species tissue-specific and common DEGs among the leaf-less control and leaf samples.


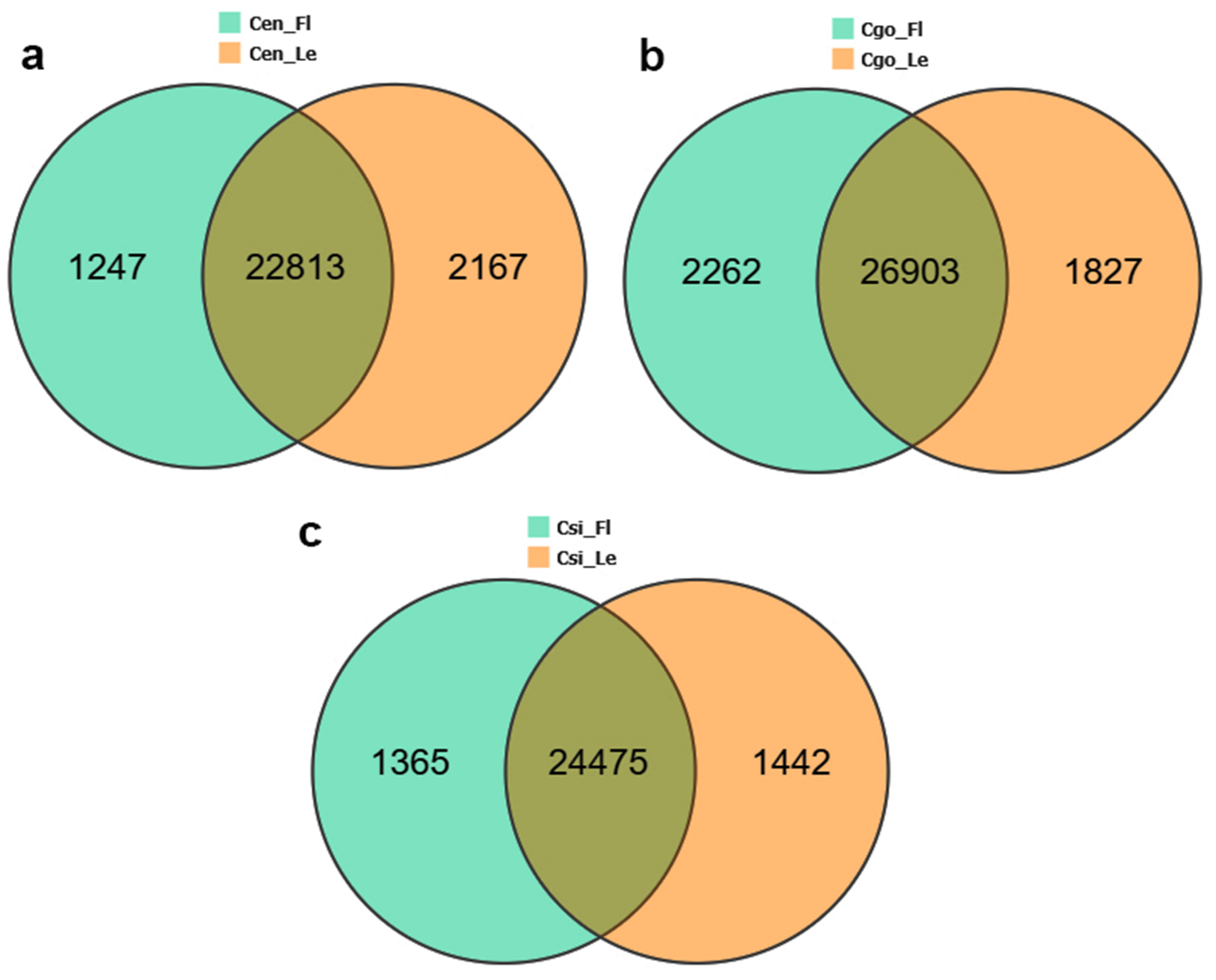


**Supplementary figure 3** Overview of GO enrichments for C. ensifolium (a), C. goeringii (b) and C. sinense (c)


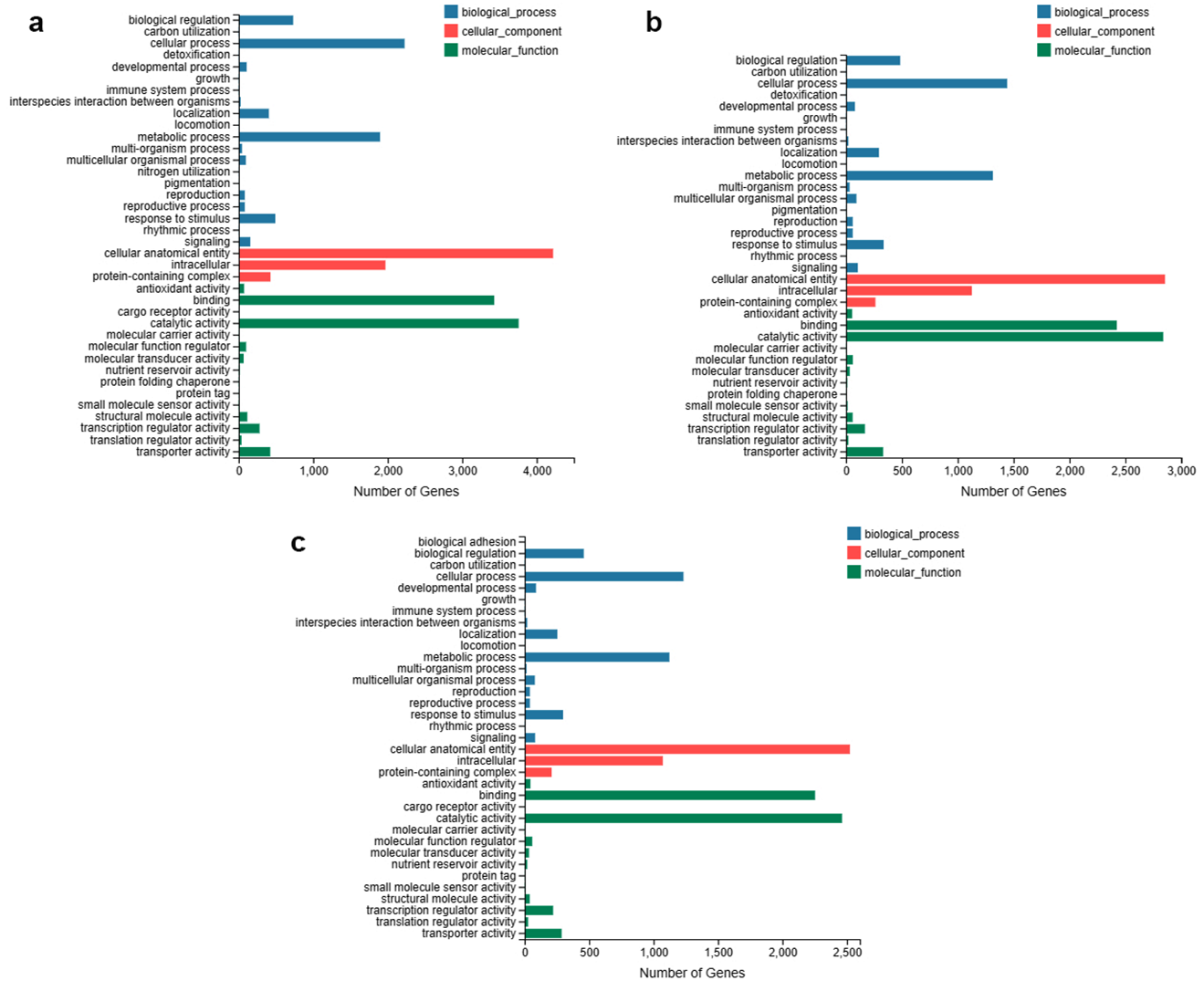


**Supplementary figure 4** Overview of KEGG enrichments for C. ensifolium (a), C. goeringii (b) and C. sinense (c)


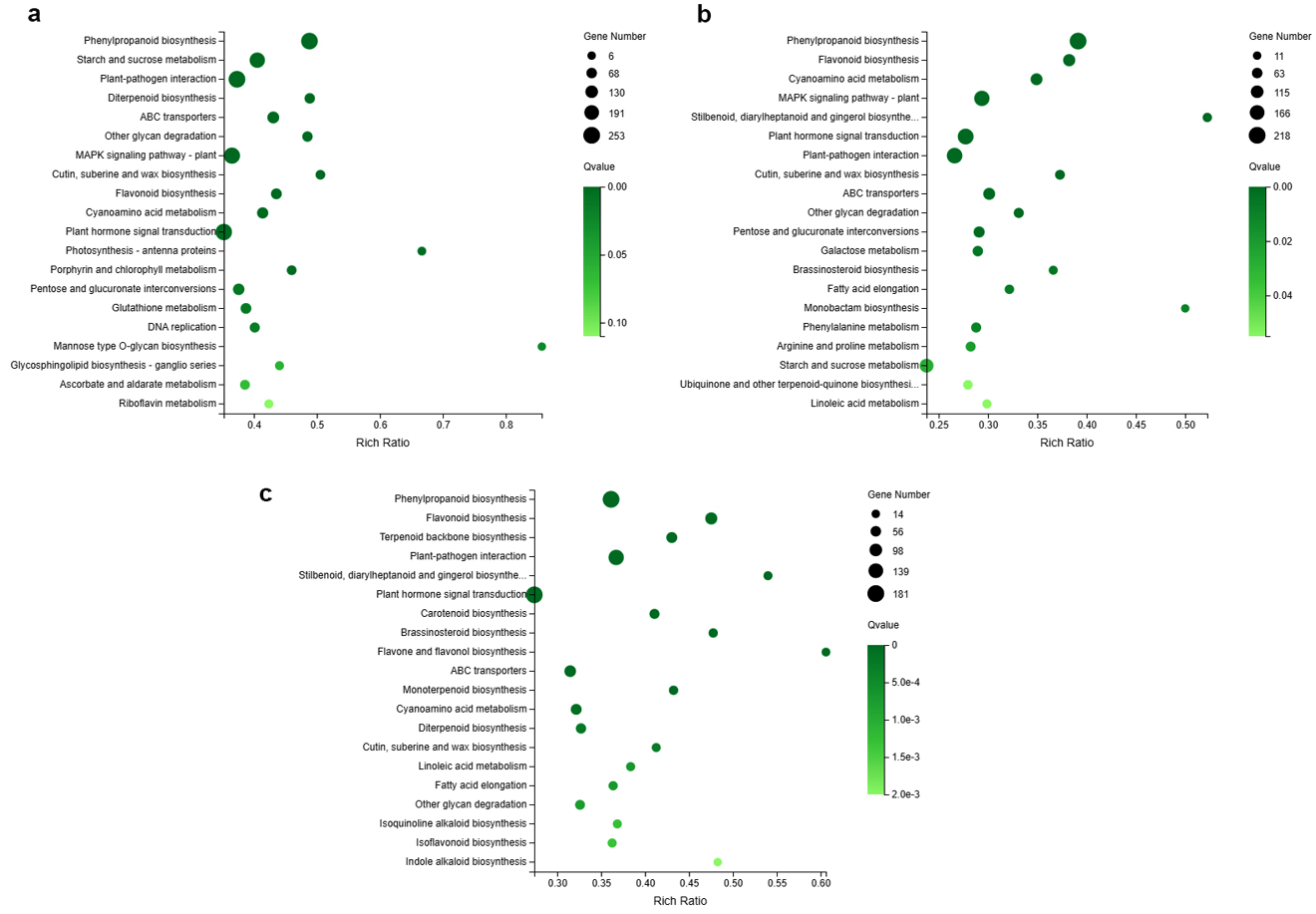

Supplement: Supplementary file 1 [file DataSheet_1.docx]
